# The study of new fixed-point iteration schemes for solving absolute value equations

**DOI:** 10.1016/j.heliyon.2024.e34505

**Published:** 2024-07-15

**Authors:** Rashid Ali, Zhao Zhang, Fuad A. Awwad

**Affiliations:** a School of Mathematical Sciences, Zhejiang Normal University, Jinhua 321004, Zhejiang, PR China; b Department of Quantitative analysis, College of Business Administration, King Saud University, P.O. Box 71115, Riyadh 11587, Saudi Arabia

**Keywords:** 90C59, 90C33, 49M20, Absolute value equations, Fixed-point schemes, Convergence theory, Numerical results

## Abstract

Absolute value equations (AVEs) play a crucial role in solving various complexities across scientific computing, engineering, management science, and operations research. This article presents two fixed-point iteration schemes designed for such AVEs. Furthermore, we show some convergence conditions under specific circumstances. Lastly, the theoretical findings are validated using numerical examples, which emphasize the distinct advantages offered by the newly developed schemes. These results are highly promising and may stimulate future research in this domain.

## 1 Introduction

Consider a matrix *A* in ${ℜ}^{n\times n}$ and a vector *b* in ${ℜ}^{n}$. We examine the AVE given by:

(1.1) As−|s|=b,

where *A* is a coefficient matrix, specifically an *M*-matrix or a strictly diagonally dominant matrix, and |s| represents a vector with elements $\left|s_{1}|,\ldots ,|s_{n}\right|$. Expanding our analysis, we delve into a more general form of AVE:

(1.2) As−B|s|=b,

where *B* is a matrix in ${ℜ}^{n\times n}$. Notably, AVE (1.2) simplifies to AVE (1.1) when $B=I_{d}$, with $I_{d}$ denoting the n×n identity matrix.

The system of AVEs constitutes a crucial class of nonlinear and non-differentiable systems with widespread applicability across diverse domains. This distinctive characteristic is encountered in various fields, including bimatrix games, contact problems, linear and convex quadratic programming, and network pricing, among others (refer to [1], [2], [3], [4], [5], [6], [7] for detailed discussions). Furthermore, the importance and utility of developing numerical algorithms and theoretical frameworks for AVEs go beyond their theoretical implications, encompassing a vast array of potential applications as well as substantial economic value. Therefore, addressing the challenges posed by AVEs is not only theoretically valuable but is also economically significant.

The study of numerical approaches for AVEs involves a comprehensive investigation into solution structures, mathematical hypotheses, algebraic frameworks, and the distinctive implementation of robust preconditioners and high-performance numerical algorithms. In recent periods, there has been a notable increase in interest in numerical procedures for AVEs, with many research publications offering different strategies. The growing attraction highlights the significance of refining strategies and processes for effectively managing the complexities inherent in AVEs. The evolving landscape of numerical strategies reflects a heightened focus on understanding solution features and underlines the continuous pursuit of innovative procedures to compute the challenges raised by AVEs. For example, Salkuyeh [8] familiarized the Picard-HSS approach for AVEs and discussed its convergence states. Khan et al. [9] have offered a new technique for AVEs based on a generalized Newton's technique and Simpson's rule. Chen et al. [10] contributed by suggesting exact and inexact Douglas–Rachford splitting procedures tailored for computing large-scale sparse AVEs, escorted by a thorough exploration of significant convergence theorems appropriate to their study. Noor et al. [11] have presented minimization strategies for AVEs and analyzed the convergence of these strategies under reasonable circumstances. Iqbal et al. [12] offered the Levenberg-Marquardt method for AVEs and examined the convergence effects in detail. Abdallah et al. [13] reformulated the AVE problem as a linear complementarity problem (LCP) and used a smoothing strategy to compute solutions. Li [14] introduced an innovative preconditioner AOR (accelerated over-relaxation) approach to solve AVEs. Prokopyev [15] studied the unique characteristics of AVEs and their affinity with LCPs. Ke and Ma [16] proposed an SOR-like approach for AVEs and discussed its convergence in detail. Based on this, Chen et al. [17] thoroughly examined the approach proposed in [16] and proposed optimal parameters for an SOR-like system. Fakhazadh and Shams [18] introduced a mixed-type splitting method for calculating AVE solutions and examined their convergence effects. Prior analysis by Zhao and Shao [19] familiarized a matrix-splitting iteration method for generalized AVEs, explaining the convergence aspects of their suggested approach. Ali and Pan [20] proposed a novel generalized iteration methodology tailored for AVEs, furnishing an insightful theoretical conversation of their analysis. Hladík and Moosaei [21] investigated AVE solvability conditions. By delving into the existing notes on solvability necessities, they also delivered valuable insights by showing two distinct generalizations of sufficient conditions that ensure AVE solvability. Zhou et al. [22] introduced an innovative strategy for solving generalized AVEs utilizing a modified Newton-based matrix splitting iteration. Their investigation contained a detailed analysis of aspects such as convergence rate, stability, and robustness across a variety of scenarios. In addition, Zhang et al. [23] introduced a newly devised two-sweep iteration strategy, which further boosts the range of techniques available for addressing AVEs.

In recent years, Dehghan and Hajarian [24], Mao et al. [25], and Li et al. [26] have designed various approaches to computing LCPs utilizing fixed-point algorithms. This investigation aims to extend this strategy to the realm of AVEs by leveraging the fixed-point principle and formulating useful schemes for solving AVE (1.1). The key contributions of this work can be sketched as follows: firstly, we decompose matrix *A* into three distinguishable parts (diagonal, strictly upper and lower triangular matrices), integrating them into the fixed-point formula to emanate novel iterative schemes. In addition, we research and establish the convergence properties of these newly formulated schemes in a variety of contexts.

The paper is structured as follows: Section 2 discusses the analysis and design of new schemes for computing AVE (1.1) and examines their convergence results. In Section 3, numerical simulation results are presented along with a detailed analysis of outcomes and a validation of the proposed methodologies. In Section 4, conclusions are drawn based on a comprehensive analysis of the previous sections, demonstrating implications and emphasizing the general relevance of the work.

## 2 Proposed schemes

This section explains the proposed schemes for solving AVE (1.1). First, we will discuss several preliminary findings in order to lay the groundwork for the rest of the discussion.

Throughout this analysis, the symbols ρ(A), Tdg(A), and $\left|A|=(|a_{ij}|\right)$ signify the spectral radius, tridiagonal matrix, and absolute value of matrix *A*, respectively. The matrix $A=(a_{ij})\in {ℜ}^{n\times n}$ is termed a *Z*-matrix if its off-diagonal components are exclusively nonpositive. If *A* is a *Z*-matrix and is nonsingular with $A^{-1}\geq 0$, it is termed as an *M*-matrix. Additionally, for each i=1,2,…,n, the matrix *A* is considered strictly row diagonally dominant when the inequality $\sum_{j=1,j\neq i}^{n}|a_{ij}|<|a_{ii}|$ is satisfied.

In order to introduce new schemes, we split matrix *A* into the following structure:

(2.3) A=D−U−L=(Q+D−U)−(Q+L)=Ω−N,

where D, L, and U stand as the diagonal, strictly lower-triangular, and strictly upper-triangular matrices, respectively. Accompanying them is Q, which is $Q=diag(\mu_{1},\mu_{2},\mu_{3},...,\mu_{n})\in {ℜ}^{n\times n}$ with 0<μ≤1. Furthermore, we assumed that Ω=Q+D−U and N=Q+L as stated in equation (2.3). The research conducted in [27], [28] demonstrated an equivalent transformation of the AVE (1.1) into a fixed point system of equations, given by

(2.4) s=s−μE[As−|s|−b],

with *μ* as a positive constant and *E* representing a diagonal matrix with positive diagonal elements. Upon selecting $E=D^{-1}$ (see for more details [29], [30]), the expression in equation (2.4) can be expressed in the following manner:

(2.5) $$s=s-\mu D^{-1}[As-|s|-b].$$

As a result of combining equations (2.3) and (2.5), we obtain the following results:

$$s=s-\mu D^{-1}[\Omega s-Ns-|s|-b],$$

or, equivalently,

$$(I_{d}-\mu D^{-1}N)s=s-\mu D^{-1}[\Omega s-|s|-b],$$

where $I_{d}$ is the identity matrix. Now we can express our iterative scheme, denoted as ***Scheme I***, for AVE (1.1) as

(2.6) $$s^{i+1}={\left(I_{d}-\mu D^{-1}N\right)}^{-1}\{s^{i}-\mu D^{-1}[\Omega s^{i}-|s^{i}|-b]\},\;i=0,1,2,\ldots .$$

Next, we suppose the convergence of *Scheme I*.

Theorem 2.1 *Consider the sequence* $\left\{s^{i}\right\}$ *generated using the procedure described by formula* (2.6) *in Scheme I, and let* $s^{⁎}$ *represent the solution of AVE* (1.1) *. Then, the following inequality holds:*

$$|s^{i+1}-s^{⁎}|\leq |W^{-1}|P|s^{i}-s^{⁎}|,$$

*where* $W=I_{d}-\mu D^{-1}N$ *and* $P=\mu D^{-1}+|I_{d}-\mu D^{-1}\Omega |$ *. Additionally, if* $\rho (|W^{-1}|P)<1$ *, the sequence* $\left\{s^{i}\right\}$ *converges to the unique solution* $s^{⁎}$ *of AVE* (1.1) *.*

Proof Consider $s^{⁎}$ be a solution of equation (1.1). In this case,

(2.7) $$s^{⁎}={\left(I_{d}-\mu D^{-1}N\right)}^{-1}\{s^{⁎}-\mu D^{-1}[\Omega s^{⁎}-|s^{⁎}|-b]\}.$$

Upon subtracting equation (2.7) from equation (2.6), the resulting expression is given by

$$s^{i+1}-s^{⁎}={\left(I_{d}-\mu D^{-1}N\right)}^{-1}\{(s^{i}-s^{⁎})-\mu D^{-1}\Omega (s^{i}-s^{⁎})+\mu D^{-1}(|s^{i}|-|s^{⁎}|)\},$$

or

$$s^{i+1}-s^{⁎}={\left(I_{d}-\mu D^{-1}N\right)}^{-1}\{(I_{d}-\mu D^{-1}\Omega )(s^{i}-s^{⁎})+\mu D^{-1}(|s^{i}|-|s^{⁎}|)\}.$$

By applying the absolute value to each side, we subsequently deduce the inequalities

$$|s^{i+1}-s^{⁎}|\leq |{\left(I_{d}-\mu D^{-1}N\right)}^{-1}|\{|I_{d}-\mu D^{-1}\Omega ||s^{i}-s^{⁎}|+\mu D^{-1}||s^{i}|-|s^{⁎}||\},$$

$$|s^{i+1}-s^{⁎}|\leq |{\left(I_{d}-\mu D^{-1}N\right)}^{-1}|\{|I_{d}-\mu D^{-1}\Omega ||s^{i}-s^{⁎}|+\mu D^{-1}|s^{i}-s^{⁎}|\},$$

$$|s^{i+1}-s^{⁎}|\leq |{\left(I_{d}-\mu D^{-1}N\right)}^{-1}|\{(|I_{d}-\mu D^{-1}\Omega |+\mu D^{-1})|s^{i}-s^{⁎}|\},$$

or, equivalently, as

$$|s^{i+1}-s^{⁎}|\leq |{\left(I_{d}-\mu D^{-1}N\right)}^{-1}|\{(\mu D^{-1}+|I_{d}-\mu D^{-1}\Omega |)|s^{i}-s^{⁎}|\}.$$

It follows that

$$|s^{i+1}-s^{⁎}|\leq |W^{-1}|P|s^{i}-s^{⁎}|.$$

Note that the matrix $|W^{-1}|P$ is nonnegative. As stated in references [29], [30], when $\rho (|W^{-1}|P)<1$, the iterative sequence $\left\{s^{i}\right\}$ generated by *Scheme I* converges towards the solution $s^{⁎}$ of the AVE (1.1).

To specify the uniqueness of the solution, assume that $t^{⁎}$ is another solution to AVE (1.1). This implies that;

$$As^{⁎}-|s^{⁎}|=b,$$

and

$$At^{⁎}-|t^{⁎}|=b,$$

which we rewrite as

$$s^{⁎}={\left(I_{d}-\mu D^{-1}N\right)}^{-1}\{s^{⁎}-\mu D^{-1}[\Omega s^{⁎}-|s^{⁎}|-b]\},$$

and

$$t^{⁎}={\left(I_{d}-\mu D^{-1}N\right)}^{-1}\{t^{⁎}-\mu D^{-1}[\Omega t^{⁎}-|t^{⁎}|-b]\},$$

respectively. It follows that

$$|s^{⁎}-t^{⁎}|\leq |W^{-1}|P|s^{⁎}-t^{⁎}|,$$

where $W=I_{d}-\mu D^{-1}N$ and $P=\mu D^{-1}+|I_{d}-\mu D^{-1}\Omega |$. Since $\rho (|W^{-1}|P)<1$, this implies that $s^{⁎}=t^{⁎}$. This concludes the proof. □

Next, we will examine ***Scheme II***. Recall that the AVE (1.1) can be restated as expressed in equation (2.5):

$$s=s-\mu D^{-1}[As-|s|-b],$$

or, equivalently, as

(2.8) $$s=\mu \{s-D^{-1}[As-|s|-b]\}+(1-\mu )s.$$

Let $\Gamma =\alpha I_{d}$, where $I_{d}$ represents the identity matrix, and 0<α≤1. Equations (2.3) and (2.8) together imply the following successively:

$$s=\mu \{s-D^{-1}[(\Omega +\Gamma )s-(N+\Gamma )s-|s|-b]\}+(1-\mu )s,$$

$$s=\mu \{s-D^{-1}[(\Omega +\Gamma )s-(N+\Gamma )s-|s|-b]\}+(1-\mu )s,$$

$$s-\mu D^{-1}(N+\Gamma )s=\mu \{s-D^{-1}[(\Omega +\Gamma )s-|s|-b]\}+(1-\mu )s,$$

and finally

$$(I_{d}-\mu D^{-1}(N+\Gamma ))s=\mu \{s-D^{-1}[(\Omega +\Gamma )s-|s|-b]\}+(1-\mu )s.$$

The iterative sequence developed by our ***Scheme II*** for computing AVE (1.1) is represented by

(2.9) $$s^{i+1}={\left(I_{d}-\mu D^{-1}(N+\Gamma )\right)}^{-1}\{\mu \{s^{i}-D^{-1}[(\Omega +\Gamma )s^{i}-|s^{i}|-b]\}+(1-\mu )s^{i}\},\;\;i=0,1,2,\ldots .$$

Next, we employ the subsequent theorem to examine the convergence of *Scheme II*.

Theorem 2.2 *Consider the iterative sequences denoted by*$\left\{s^{i}\right\}$*generated through Scheme* II*, and let* $s^{⁎}$ *represent the solution to AVE* (1.1)*. In this context,*

$$|s^{i+1}-s^{⁎}|\leq |J^{-1}|F|s^{i}-s^{⁎}|,$$

*where* $J=I_{d}-\mu D^{-1}(N+\Gamma )$ *and* $F=\mu D^{-1}+|I_{d}-\mu D^{-1}(\Omega +\Gamma )|$*. Additionally, if* $\rho (|J^{-1}|F)<1$*, then the sequence* $\left\{s^{i}\right\}$ *converges to the unique solution* $s^{⁎}$ *of the AVE* (1.1)*.*

Proof Consider $s^{⁎}$ be a solution of equation (1.1). Then

(2.10) $$s^{⁎}={\left(I_{d}-\mu D^{-1}(N+\Gamma )\right)}^{-1}\{\mu \{s^{⁎}-D^{-1}[(\Omega +\Gamma )s^{⁎}-|s^{⁎}|-b]\}+(1-\mu )s^{⁎}\}.$$

By deducting equation (2.10) from equation (2.9), we obtain

$$s^{i+1}-s^{⁎}={\left(I_{d}-\mu D^{-1}(N+\Gamma )\right)}^{-1}\{\mu \{(s^{i}-s^{⁎})-D^{-1}(\Omega +\Gamma )(s^{i}-s^{⁎})+D^{-1}(|s^{i}|-|s^{⁎}|\}+(1-\mu )(s^{i}-s^{⁎})\}.$$

As a result of simplifying the right-hand side, we obtain

$$s^{i+1}-s^{⁎}={\left(I_{d}-\mu D^{-1}(N+\Gamma )\right)}^{-1}\{(I_{d}-\mu D^{-1}(\Omega +\Gamma ))(s^{i}-s^{⁎})+\mu D^{-1}(|s^{i}|-|s^{⁎}|)\}.$$

By applying the absolute values on both sides of the equation, we successively acquire

$${\left|s^{i+1}-s^{⁎}|\leq |I_{d}-\mu D^{-1}(N+\Gamma )\right)}^{-1}|\{|I_{d}-\mu D^{-1}(\Omega +\Gamma )||s^{i}-s^{⁎}|+\mu D^{-1}||s^{i}|-|s^{⁎}||\},$$

hence

$${\left|s^{i+1}-s^{⁎}|\leq |I_{d}-\mu D^{-1}(N+\Gamma )\right)}^{-1}|\{|I_{d}-\mu D^{-1}(\Omega +\Gamma )||s^{i}-s^{⁎}|+\mu D^{-1}|s^{i}-s^{⁎}|\},$$

or

$${\left|s^{i+1}-s^{⁎}|\leq |I_{d}-\mu D^{-1}(N+\Gamma )\right)}^{-1}|(\mu D^{-1}+|I_{d}-\mu D^{-1}(\Omega +\Gamma )|)|s^{i}-s^{⁎}|,$$

ending with

$$|s^{i+1}-s^{⁎}|\leq |J^{-1}|F|s^{i}-s^{⁎}|,$$

where

$$J=I_{d}-\mu D^{-1}(N+\Gamma )\;\mathrm{and}\;F=\mu D^{-1}+|I_{d}-\mu D^{-1}(\Omega +\Gamma )|.$$

Certainly, if the condition $\rho (|J^{-1}|F)<1$ is satisfied, the sequence of iterations $\left\{s^{i}\right\}$ formed by *Scheme II* converges.

The demonstration of uniqueness for *Scheme II* follows a similar explanation as the proof presented in Theorem 2.1 and is excluded from the discussion. □

## 3 Numerical tests

Here, we present findings from a variety of numerical analyses illustrating the effectiveness of the developed schemes. The assessments focus on factors such as the number of iteration steps (referred to as **IT**), CPU time (referred to as **CPU**), and relative residual error (referred to as **RES**). The RES is represented by the expression:

$$\mathrm{RES}:=\frac{{\left\|As^{i}-|s^{i}|-b\right\|}_{2}}{{\left\|b\right\|}_{2}},$$

and RES is constrained by the condition $\mathrm{RES}\leq 10^{-6}$. We conducted our simulations utilizing MATLAB 2016a on a system equipped with an Intel(R) Core(TM) i5-3337U CPU 1.80 GHz and 4.00 GB RAM.

Initially, we conduct numerical experiments to meet the convergence criteria, specifically ensuring that both $\rho (|W^{-1}|P)<1$ and $\rho (|J^{-1}|F)<1$. Table 1 shows the outcomes of these analyses.

**Table 1:** Convergence requirement prescribed by Theorem 2.1, Theorem 2.2.

Examples	*n*	*Scheme I*	*Scheme II*
		$\rho (|W^{-1}|P)$	$\rho (|J^{-1}|F)$
Ex. 3.1	100	0.2575	0.4937
	4900	0.2958	0.5646
Ex. 3.2	256	0.1721	0.2623
	2401	0.1764	0.2678
Ex. 3.3	3000	0.3322	0.3321
	5000	0.3327	0.3325

Based on Table 1, we conducted numerical investigations to assess the convergence conditions for both theorems. The study found that both schemes perform effectively and satisfy the convergence conditions. To thoroughly evaluate the implementation of our developed schemes, we carried out the following tests.

Example 3.1 ([18]) Let *ϑ* be a given positive integer, and set $n=\vartheta^{2}$. Consider the AVE (1.1), in which $A\in {ℜ}^{n\times n}$ to be of the form $A=M+I_{d}$, where

$$M=\mathrm{Tdg}(-1.5I_{d},S,-0.5I_{d})=\left(\begin{array}{cccccc} S & -0.5I_{d} & & & & \\ -1.5I_{d} & S & -0.5I_{d} & & & \\ & \ddots & S & \ddots & & \\ & & \ddots & \ddots & -0.5I_{d} & \\ & & & -1.5I_{d} & S & \end{array}\right)\in {ℜ}^{n\times n},$$

is a block-tridiagonal matrix, and

$$S=\mathrm{Tdg}(-1.5,4,-0.5)=\left(\begin{array}{cccccc} 4 & -0.5 & & & & \\ -1.5 & 4 & -0.5 & & & \\ & \ddots & 4 & \ddots & & \\ & & \ddots & \ddots & -0.5 & \\ & & & -1.5 & 4 & \end{array}\right)\in {ℜ}^{\vartheta \times \vartheta },$$

and

$$b=As^{⁎}-|s^{⁎}|\;\text{with}\;s^{⁎}={\left(-1,2,1,2,...,1,2\right)}^{T}\in {ℜ}^{n}.$$

**Figure 1:**
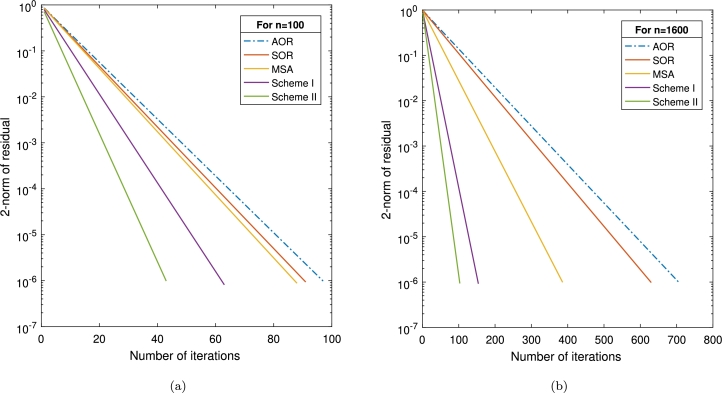
Graphical representation of various schemes in Example 3.1 with (a) *n* = 100 and (b) *n* = 1600.

Here, we consider the effectiveness of the proposed schemes I and II in comparison with three existing approaches: the AOR and SOR approaches [14] and the mixed-type splitting approach (MSA) [18]. A summary of the comparison can be found in Table 2. Moreover, we consider problem sizes of n=100 and n=1600 and compare all strategies graphically. A graphic expression of the outcomes can be found in Fig. 1.

**Table 2:** The results of Example 3.1 when employing *μ* = 0.9 and *α* = 0.8.

Schemes	n	100	400	900	1600	4900
	IT	97	190	336	706	784
*AOR*	CPU	0.6729	3.0199	4.5381	5.7266	9.0295
	RES	9.80e–07	9.61e–07	9.73e–07	9.84e–07	9.36e–07

	IT	91	178	296	630	671
*SOR*	CPU	0.4217	2.6311	3.0470	4.7008	5.0912
	RES	9.38e–07	9.11e–07	9.48e–07	9.64e–07	9.95e–07

	IT	88	157	250	386	392
*MSA*	CPU	0.3321	1.7993	2.0275	3.0264	5.0048
	RES	8.91e–07	9.65e–07	9.18e–07	9.56e–07	9.89e–07

	IT	63	97	127	154	183
*Scheme I*	CPU	0.2835	1.0177	1.9221	2.2472	3.9201
	RES	8.17e–07	9.52e–07	9.03e–07	8.60e–07	8.72e–07

	IT	43	66	85	103	114
*Scheme II*	CPU	0.1429	0.3932	0.9161	1.8021	2.3827
	RES	9.84e–07	9.60e–07	9.93e–07	9.39e–07	9.18e–07

Table 2 provides a comprehensive analysis of the solution $s^{⁎}$ across various *n* values for each procedure. The results demonstrate that the schemes we introduced exhibit superior effectiveness compared to AOR, SOR, and MSA, especially in terms of both “IT” and “CPU”.

Furthermore, we have included the convergence curves for all methodologies that have been implemented using the size of the problems of n=100 and n=1600 as well. As a result, the curves in Fig. 1 indicate the superiority of our new schemes over those of other methods.

**Figure 2:**
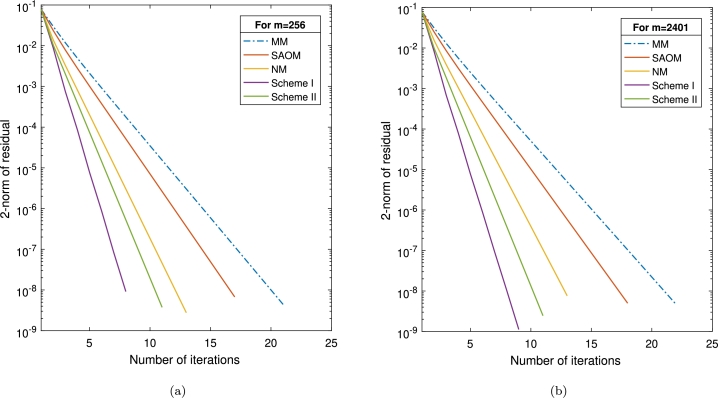
Graphical representation of various schemes in Example 3.2 with (a) *n* = 256 and (b) *n* = 2401.

Example 3.2 ([27]) Consider the matrix *A*, defined as $A=I_{d}⊗\nabla +\Theta ⊗I_{d}\in {ℜ}^{m\times m}$, where $I_{d}$ represents the identity matrix in ${ℜ}^{m\times m}$, and ⊗ denotes the Kronecker product. Additionally, the matrices ∇ and Θ are tridiagonal with dimensions n×n, defined as follows:

$$\left\{ \begin{array}{c} \nabla =\mathrm{Tdg}[(\frac{2+\gamma }{8}),8,(\frac{2-\gamma }{8})],\\ \Theta =\mathrm{Tdg}[(\frac{1+\gamma }{4}),4,(\frac{1-\gamma }{4})], \end{array}\right.$$

where $\gamma =\frac{1}{n}$ and $m=n^{2}$. The vector *b* is expressed as $b=As^{⁎}-|s^{⁎}|$, with $s^{⁎}=\text{ones}(m,1)\in {ℜ}^{m}$. We employ identical stopping criteria and an initial vector as specified in the reference [27]. To evaluate the efficacy of our suggested schemes, we execute a comparative analysis against the approach presented in [27] (displayed as *MM*), the SOR-like approximate optimal method (*SAOM*) [17], and the method outlined in [31] (referred to as *NM*). The outcomes of these comparisons are meticulously studied and reported in Table 3. Additionally, we explore problem sizes of m=256 and m=2401, conducting a graphical comparison of all strategies. The visual expression of the results is presented in Fig. 2.

**Table 3:** The results of Example 3.2 when employing *μ* = 1 and *α* = 0.8.

Schemes	*m*	256	1296	2401	4096
	IT	21	21	22	23
*MM*	CPU	0.9372	2.0279	5.3933	7.8106
	RES	4.42e–09	3.20e–09	4.63e–09	5.64e–09

	IT	17	18	18	18
*SAOM*	CPU	0.6204	1.9734	4.7935	6.6510
	RES	6.81e–09	3.74e–09	5.08e–09	6.62e–09

	IT	13	13	13	14
*NM*	CPU	0.5134	1.1958	4.0818	5.5514
	RES	2.75e–09	5.75e–09	7.86e–09	1.58e–09

	IT	8	9	9	9
*Scheme I*	CPU	0.2378	0.6049	2.2383	3.6474
	RES	9.13e–09	1.05e–09	1.14e–09	1.12e–09

	IT	11	11	11	11
*Scheme II*	CPU	0.2569	0.8247	2.9318	4.0148
	RES	3.72e–09	1.28e–09	2.26e–09	2.46e–09

Table 3 illustrates the effectiveness and accuracy of various methods of addressing the given problem. Upon closer examination, it is evident that *Scheme I* achieves superior results to all other techniques, including *Scheme II*. Furthermore, our proposed schemes, namely I and II, consistently produce superior results when compared to alternative methods, particularly in terms of both “IT” and “CPU” considerations.

Moreover, we have incorporated convergence plots for all methodologies utilized, assuming problem sizes of m=256 and m=2401. The graphs in Fig. 2 vividly demonstrate the superior performance of our innovative schemes compared to the MM, SAOM, and NM methods.

Example 3.3 ([11]) Consider the square matrix $A={\left(a_{ij}\right)}_{n\times n}$, where each element is defined as follows:

$$a_{ii}=4n,\;a_{i,i+1}=a_{i+1,i}=n,\;a_{ij}=0.5\;\;(i=1,2\ldots ,n).$$

Define the vector *b* as $b=(A-I_{d})e$, where $I_{d}$ is the identity matrix of order *n*, and *e* is an n×1 vector with all elements equal to unity. Notably, the actual solution is denoted by $s={\left(1,1,\ldots ,1\right)}^{T}$.

**Figure 3:**
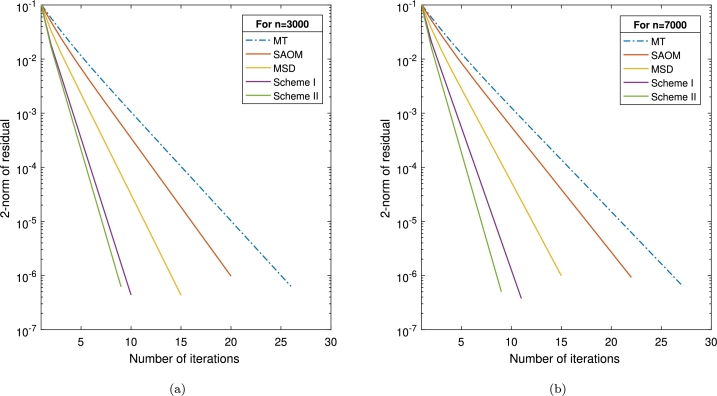
Graphical representation of various schemes in Example 3.3 with (a) *n* = 3000 and (b) *n* = 7000.

For this example, we initialize the vector as $s^{\left(0\right)}={\left(s_{1},s_{2},\ldots ,s_{j}\right)}^{T}$, with $s_{j}=0.001⁎j$. We compare the proposed techniques with the minimization technique from [11] (referred to as MT), the SOR-like approximate optimal method (*SAOM*) [17], and the modified search direction iteration method from [32] (specified as MSD). An overview of the results is given in Table 4, and a graphic representation of n=3000 and n=7000 can be found in Fig. 3.

**Table 4:** The results of Example 3.3 when employing *μ* = 1 and *α* = 0.8.

Schemes	*n*	3000	4000	5000	6000	7000
	IT	26	27	27	27	27
*MT*	CPU	1.9212	3.5137	7.3724	11.4393	14.2520
	RES	5.73e–07	4.52e–07	7.41e–07	5.68e–07	6.91e–07

	IT	20	21	21	22	22
*SAOM*	CPU	0.9228	2.5138	4.2291	4.9094	6.0209
	RES	9.04e–07	7.01e–07	9.60e–07	6.12e–09	9.28e–07

	IT	15	15	15	15	15
*MSD*	CPU	0.8184	2.0718	3.4149	4.6431	5.4396
	RES	4.23e–07	3.62e–07	7.58e–07	3.68e–07	9.88e–07

	IT	10	10	10	11	11
*Scheme I*	CPU	0.6488	0.9231	1.5837	2.5418	3.8575
	RES	4.31e–07	6.96e–07	9.63e–07	2.73e–07	3.69e–07

	IT	9	9	9	9	9
*Scheme II*	CPU	0.5151	0.7092	1.3371	2.2971	2.8510
	RES	6.008e–07	7.17e–7	2.49e–07	3.11e–07	4.99e–07

Based on the outcomes given in Table 4, all of the tested schemes in the study demonstrate a rapid ability to test the system solution (1.1). Notably, our proposed schemes showcase fewer iterations (IT) and reduced analysis time (CPU) compared to alternative techniques. We have also included convergence graphs for all methodologies, with problem sizes set at n=3000 and n=7000. The visuals in Fig. 3 compellingly demonstrate the superior efficacy of our inventive strategies when contrasted with the MT, SAOM, and MSD methods. In summary, this finding strongly supports the feasibility and significant advantages of the suggested schemes for AVEs.

## 4 Conclusions

In the article, we have presented two innovative iterative schemes designed to compute solutions to equation (1.1). In addition, we have examined the convergence theorems for the suggested schemes. Finally, several numerical analyses have been described, demonstrating the applicability and effectiveness of the proposed schemes.

In this analysis, our investigation specifically focused on AVEs where the coefficient matrix adheres to the properties of an *M*-matrix or a strictly diagonally dominant matrix. We intend to focus our future studies on AVEs with various broader classes of coefficient matrices.

## Funding

This project is funded by 10.13039/501100002383King Saud University, Riyadh, Saudi Arabia, and 10.13039/501100004706Zhejiang Normal University, China.

## CRediT authorship contribution statement

**Rashid Ali:** Writing – review & editing, Writing – original draft, Visualization, Validation, Software, Resources, Methodology, Investigation, Formal analysis, Data curation, Conceptualization, Supervision. **Zhao Zhang:** Writing – review & editing, Resources, Methodology, Investigation, Data curation, Validation. **Fuad A. Awwad:** Writing – review & editing, Writing – original draft, Validation, Resources, Project administration, Funding acquisition.

## Declaration of Competing Interest

The authors declare that they have no known competing financial interests or personal relationships that could have appeared to influence the work reported in this paper.

## Data Availability

Data will be made available on request.
